# Birth Weight, Maternal Body Mass Index, and Early Childhood Growth: A Prospective Birth Cohort Study in China

**DOI:** 10.2188/jea.JE20090187

**Published:** 2010-11-05

**Authors:** Rongwei Ye, Lijun Pei, Aiguo Ren, Yali Zhang, Xiaoying Zheng, Jian-meng Liu

**Affiliations:** 1 Institute of Reproductive and Child Health, Peking University Health Science Center, Beijing, PR China; 2 Department of Epidemiology and Biostatistics, School of Public Health, Peking University Health Science Center, Beijing, PR China; 3 Peking University Institute of Population Research, Beijing, PR China

**Keywords:** birth weight, overweight, underweight, maternal body mass index

## Abstract

**Background:**

The relations of birth weight and maternal body mass index (BMI) to overweight remain unresolved. We prospectively examined the relations of birth weight with various anthropometric measures at age 3 to 6 years, the effect of maternal BMI, and the patterns of these relations in an analysis using 9 birth weight categories.

**Methods:**

The subjects were 210 172 singleton infants born alive with a gestational age ≥28 weeks between October 1993 and December 1996; the subjects were followed up in 2000. Birth weight, maternal height and weight, and other relevant information were measured or collected prospectively. Overweight and underweight were defined by using National Center for Health Statistics/World Health Organization reference data. Logistic regression models were used to estimate relative risks. Analyses stratified by quartile of maternal BMI were performed to examine the effects of maternal BMI on the associations of birth weight with overweight and underweight.

**Results:**

Birth weight was linearly associated with height, weight, and BMI at age 3–6 years. Adjustment for maternal BMI did not alter this association. Birth weight was positively associated with overweight and negatively associated with underweight. The relation curves for both overweight and underweight resembled half of a flat parabolic curve. The associations for overweight and underweight were slightly stronger for the highest and lowest quartiles of maternal BMI, respectively.

**Conclusions:**

Higher birth weight is associated with an increased risk for childhood overweight, and lower birth weight with an increased risk for underweight. The associations between birth weight and early childhood anthropometric growth measures could not explained by maternal BMI.

## INTRODUCTION

Childhood obesity is associated with increased risks for several adult chronic diseases. This is of great concern, as the number of overweight children is growing worldwide.^1^^–^^3^ Fetal growth in the uterus is crudely indicated by birth weight, and affects growth later in life.^4^^,^^5^ Epidemiological studies have focused on the association of birth weight to overweight/obesity in childhood^6^^,^^7^ and adulthood.^8^^,^^9^ Several longitudinal studies have found a positive association between birth weight and overweight in both children and adults, without extensive adjustment for potential confounders such as maternal body mass index (BMI), gestational age at birth, pregnancy-induced hypertension, maternal weight gain, maternal smoking, or alcohol consumption during pregnancy.^10^ In addition, the relationships among birth weight, maternal BMI, and overweight remain unresolved.^11^ Some studies reported^12^ that the positive relation between birth weight and overweight in later life only existed in some strata of maternal BMI; others reported^13^ that maternal BMI was a more important risk factor than birth weight for childhood overweight.

A few studies^14^ have prospectively examined the relations of birth weight with mean height, weight, and BMI measured before age 7 years. The 1958 British birth cohort^13^ comprised 10 683 participants and reported that the curve for the relation between birth weight and BMI at age 7 years was weakly linear; however, it was J-shaped for BMI at age 33 years, when birth weight categories were restricted to tertile cut-offs, probably due to small sample size.

The data are limited regarding the relation between birth weight and low anthropometric indices such as childhood underweight. Low anthropometric indices are prevalent in developing countries and are associated with poor cognitive and educational performance in children.^15^ Simultaneous investigation of the associations of birth weight with overweight and underweight might aid in understanding the relation between birth weight and later growth.

Here, we attempted to comprehensively and prospectively assess the relation between birth weight and body growth, after extensive adjustment for potential confounders, in a large Chinese birth cohort followed to age 6 years. We aimed to determine if birth weight was related to underweight, as well as overweight, and if the relations persisted in all strata of maternal BMI. In addition, we generated curves for the associations of birth weight to overweight and underweight by using 9 birth weight groups.

## METHODS

### Study cohort

The subjects were 241 060 singleton infants born alive with a gestational age ≥28 weeks between October 1993 and December 1996. The mothers of the infants had all participated in a population-based prenatal care program implemented in 26 counties or cities in Zhejiang, Jiangsu, and Hebei province, China.^16^ Women entered the program either at a premarital health assessment or at any stage of pregnancy. Information about demographic and relevant obstetric and pregnancy outcomes was collected at the time of entry, subsequent prenatal visits, or delivery. All singleton children born alive to the enrolled women were followed until March through July 2000. Of the 241 060 children, 3274 children had died, 8620 had moved out of the study area, and 7532 (3.1%) were lost to follow up. After excluding 7026 children with unknown birth weight or sex and 4436 children whose height or weight were not measured at the time of follow-up, 210 172 children remained in the cohort for the primary analyses. The Peking University Health Science Center Institutional Review Board approved this study.

### Exposure

All children were born in hospitals, where birth weight was routinely assessed within 1 hour of birth and recorded. Because of the large sample size, we were able to include 9 birth weight groups (<2.50, 2.50–, 2.75–, 3.00–, 3.25–, 3.50–, 3.75–, 4.00–, ≥4.25 kg) in our investigation of the pattern of the associations between birth weight and overweight/underweight. The birth weight category of 3.00 to 3.24 kg contained the largest number of children, and was thus selected as the reference group.

### Outcomes

Childhood growth measurements included height, weight, BMI, overweight, and underweight. Height and weight were measured by trained local health workers. Weight (kg) and height (cm) were measured in light indoor clothing without shoes, overcoats, or hats. All instruments were calibrated by local experts in quality and technical supervision. BMI was calculated as weight divided by height (kg/m^2^).

Overweight was defined as Z-scores of weight-for-height >2 and underweight as Z-scores of weight-for-age ≤2, which were calculated based on National Center for Health Statistics/World Health Organization reference data using the formula, Z-score = (Observed − Median_ref_)/SD_ref_. The term overweight in this study includes both overweight and obese children.

To examine whether findings were influenced by different definitions, we further defined overweight by using international age- and sex-specific BMI cut-off points.^17^ Since the results were similar to those of the main analysis, we have not presented these data in this report.

### Potential confounding factors

Staff at the prenatal care program recorded information about maternal and infant characteristics, which enabled us to control for various potential confounding factors. Maternal factors included area of residence (northern/southern), age at delivery (<22, 22–24, 25–27, 28–30, 31–34, ≥35 years), occupation (farmer/non-farmer), education (high school or above, middle school, primary school or below), primiparous (yes/no), folic acid supplementation during pregnancy (yes/no), gestational age (<37, 37–39, 40–41, ≥42 weeks), pregnancy-induced hypertension (yes/no), and maternal weight gain during pregnancy (<P_25_ (8.0), P_25_ to <P_50_ (11.0), P_50_ to <P_75_ (14.0), P_75_ kg). Child factors included sex (male/female) and body length at birth (<P_5_ (43.0 cm), P_5_ to <P_50_ (50.0 cm), P_50_ to <P_95_ (52.0 cm), ≥P_95_). We treated all these factors as categorical variables in the analyses.

Because maternal BMI might influence the association between birth weight and overweight,^13^^,^^18^ we performed analyses stratified by maternal BMI (cut-offs for quartiles: 19.1, 20.5, and 22.0 kg/m^2^). The results showed that the effect of maternal BMI, although present, was weak; thus, maternal BMI was evaluated as a confounder.

### Statistical analyses

We used regression models to assess the associations between birth weight and early childhood growth after adjusting for covariates. In the first model, we adjusted for child residence area, sex, and age at follow-up because these 3 variables were directly and strongly associated with childhood body size in the cohort.^19^ In a subsequent model, we further adjusted for other potential confounders except maternal BMI. In the third model, we added maternal BMI.

When marginal means or relative risks were estimated, we treated birth weight and all covariates as indicators. For each birth weight category, marginal means were estimated by using general linear regression models; relative risks of overweight or underweight were estimated using logistic regression models. When testing trends, we assigned the median value or squared median value of each birth weight category as a score and treated this score as a continuous variable. When testing interactions between birth weight and maternal BMI, we included the product term of the 2 variables in a model, where only birth weight was treated as a continuous variable, using the median value of each category as a score. All analyses were performed using SPSS 11.5 (SPSS, Chicago, IL). All reported *P* values were 2-sided. A *P* value <0.05 was considered statistically significant.

## RESULTS

### Characteristics of the cohort

Among the 210 172 children in our cohort, 87.5% lived in a southern area, 51.9% were boys, and mean age (SD) at follow-up was 59.0 (8.2) months (range: 40–79 months). Overall, 99.2% of children were of Han ethnicity, the dominant ethnic group in China. A total of 63% of the mothers of the children in the cohort entered the prenatal care program during premarital health assessment, 19% during the first trimester of pregnancy, 11% during the second trimester, and 7% during the third trimester. As shown in Table 1, the mothers of children with the highest birth weights tended to have a higher mean age, mean height, mean weight, and mean BMI at first prenatal visit, and were less likely to be primiparous. Children with the highest birth weights had a higher mean gestational age and mean birth length.

**Table 1.** Characteristics of mothers and children, by selected birth weight cohort (*n* = 210 172)

Characteristics	Selected birth weight cohorts		

	<2500 g (*n* = 3911)	3000 to 3249 g (*n* = 57 885)	≥4250 g (*n* = 3048)
Mother			
Age at delivery (y)	24.5 (3.1)	24.7 (3.2)	25.7 (3.6)
Height (m)	1.57 (0.05)	1.59 (0.04)	1.61 (0.05)
Weight at first prenatal visit (kg)	49.8 (6.1)	51.5 (6.0)	56.5 (7.3)
Body mass index (kg/m^2^)	20.2 (2.3)	20.5 (2.2)	21.8 (2.6)
Residence in southern area (%)	94.4	85.7	95.1
Han ethnicity (%)	98.8	99.1	99.4
Farmer (%)	60.7	64.2	62.4
Education			
High school or above (%)	8.6	10.1	10.7
Middle school (%)	58.3	61.5	59.0
Primary school or less (%)	33.2	28.4	30.4
Folic acid supplementation before or ​ during pregnancy (%)	51.1	55.3	50.3
Primiparous (%)	86.7	85.1	71.0

Child			
Gestational age (weeks)	37.0 (2.9)	39.4 (1.7)	40.2 (1.5)
Male (%)	44.3	46.8	69.0
Birth length (cm)	46.3 (3.2)	49.3 (1.9)	51.5 (2.5)
Age at follow-up visit (months)	60.2 (8.4)	59.1 (8.2)	58.8 (8.1)

The numbers of children (median birth weight) in the 9 birth weight groups were 3911 (2.3 kg), 11 275 (2.6 kg), 19 451 (2.9 kg), 57 885 (3.1 kg), 42 058 (3.4 kg), 46 225 (3.5 kg), 16 451 (3.8 kg), 9868 (4.0 kg), and 3048 (4.4 kg).

### Relation of birth weight to mean height, weight, and BMI

**Figure 1.**
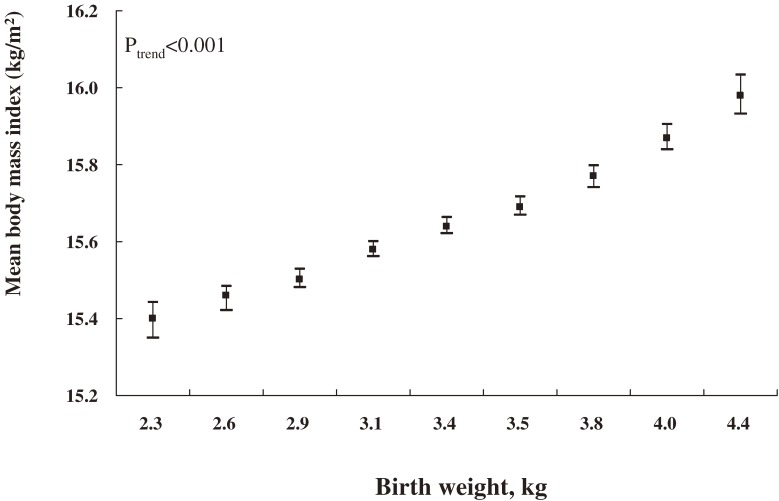
Area-, sex-, and age-adjusted means for body mass index and 95% confidence intervals (vertical lines) by birth weight for Chinese children aged 3–6 years at follow-up in 2000.

We first examined the association of birth weight with mean height, weight, and BMI, where these anthropometric measurements were treated as dependent continuous variables. The children’s area of residence, sex, and age at follow-up were known to be directly associated with their anthropometric measurements. After adjustment for these factors using generalized linear models, birth weight was positively associated with height, weight, and BMI (*P* for linear trend <0.001). Figure 1 shows means and 95% confidence intervals of BMI according to birth weight categories. The same pattern of linear relation was observed for both children’s mean height and weight (data not shown). Further adjustment for other maternal or child factors only slightly attenuated the mean estimates, but did not change the linear trend. These factors included maternal age at delivery, occupation, education, folic acid supplementation before or during pregnancy, parity, child’s gestational age, body length at birth, pregnancy-induced hypertension, and maternal weight gain during pregnancy. We also conducted analysis stratified by child’s age at follow-up, and the linear relations for children’s height, weight, and BMI were not changed in any stratum (*P* for linear trend <0.001).

Due to the positive association of maternal height and weight with childhood growth, we incorporated these variables into a model. The results were not substantially changed (Table 2). When maternal height and weight were further adjusted for, the difference in mean height between birth weight ≥4.25 and <2.50 kg decreased from 2.7 to 2.2 cm, the difference in mean weight from 1.5 to 1.3 kg, and the difference in mean BMI from 0.59 to 0.53 kg/m^2^. We also assessed the modifying effects of maternal BMI on the relation between birth weight and childhood BMI, height, and weight, but found no clear evidence that these relations were modified by maternal BMI (*P* values for the interaction tests were 0.06 for BMI, 0.64 for height, and 0.52 for weight).

**Table 2.** Multivariate-adjusted means and 95% confidence intervals for height, weight, and body mass index in Chinese children aged 3–6 years, by birth weight^a^

Birth weight (kg)	Height (cm)	Weight (kg)	Body mass index (kg/m^2^)
<2.50	103.3 (103.1–103.6)	16.5 (16.4–16.6)	15.37 (15.30–15.44)
2.50–	103.7 (103.5–103.9)	16.7 (16.6–16.8)	15.45 (15.38–15.51)
2.75–	103.9 (103.7–104.1)	16.8 (16.7–16.9)	15.49 (15.43–15.55)
3.00–	104.3 (104.1–104.5)	17.0 (16.9–17.1)	15.55 (15.49–15.61)
3.25–	104.5 (104.3–104.7)	17.1 (17.0–17.2)	15.60 (15.54–15.66)
3.50–	104.8 (104.6–105.0)	17.3 (17.2–17.3)	15.64 (15.58–15.70)
3.75–	105.2 (105.0–105.4)	17.5 (17.4–17.5)	15.71 (15.65–15.77)
4.00–	105.2 (105.0–105.5)	17.6 (17.5–17.7)	15.80 (15.74–15.87)
4.25+	105.5 (105.3–105.9)	17.8 (17.7–17.9)	15.90 (15.82–15.97)

*P* for linear trend	<0.001	<0.001	<0.001

^a^The model includes residence area (northern/southern); child’s sex (male/female); child’s age at follow-up (40–41, 42–47, 48–53, 54–59, 60–65, 66–71, 72–77, 78–79 months); maternal age at delivery (<22, 22–24, 25–27, 28–30, 31–34, ≥35 years); occupation (farmer/non-farmer); education (high school or above, middle school, primary school or below); folic acid supplementation before or during pregnancy, primiparous (yes/no); child’s gestational age at birth (<37, 37–39, 40–41, ≥42 weeks); child’s body length at birth (<P_5_ (43.0), P_5_ to <P_50_ (50.0), P_50_ to <P_95_ (52.0), ≥P_95_ cm); pregnancy-induced hypertension (yes/no); maternal weight gain during pregnancy (<P_25_ (8.0), P_25_ to <P_50_ (11.0), P_50_ to <P_75_ (14.0), ≥P_75_ kg); maternal height (<P_5_ (1.51), P_5_ to <P_25_ (1.56), P_25_ to <P_50_ (1.59), P_50_ to <P_75_ (1.61), P_75_ to <P_95_ (1.66), ≥P_95_ cm); and weight at first prenatal visit during pregnancy (<P_5_ (43.0), P_5_ to <P_25_ (48.0), P_25_ to <P_50_ (52.0), P_50_ to <P_75_ (56.0), P_75_ to <P_95_ (63.0), ≥P_95_ kg).

### Relation of birth weight to overweight and underweight

**Figure 2.**
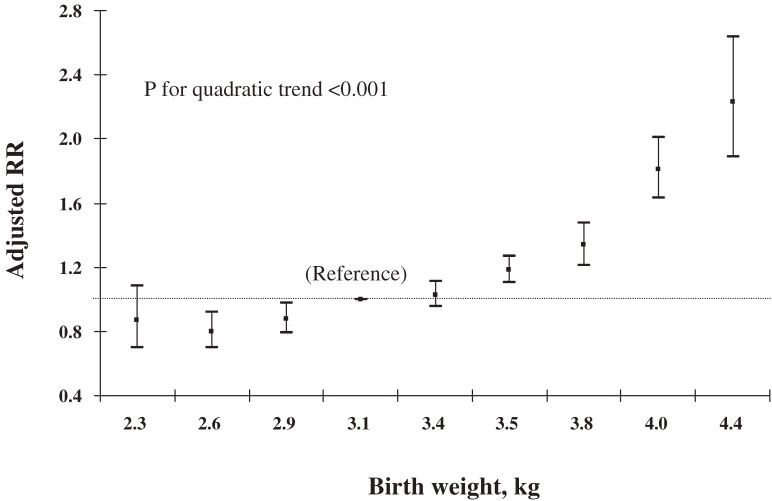
Relative risks (RRs) and 95% confidence intervals (vertical lines) for overweight by birth weight after adjustment for residence area, sex, and age of Chinese children aged 3–6 years at follow-up in 2000.

**Figure 3.**
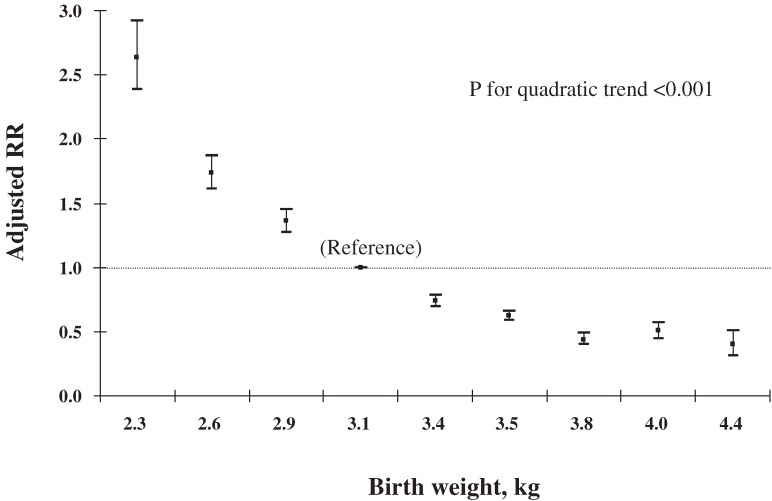
Relative risks (RRs) and 95% confidence intervals (vertical lines) for underweight by birth weight after adjustment for residence area, sex, and age of Chinese children aged 3–6 years at follow-up in 2000.

We then used logistic regression model to assess relations of birth weight to overweight and underweight. After adjustment for the child’s area of residence, sex, and age at follow-up, birth weight was positively correlated with overweight (Figure 2) and inversely correlated with underweight (Figure 3). The pattern of the association resembled the right half of a flat parabolic curve for overweight and the left half for underweight (*P* for quadratic trend <0.001). The slopes of both curves began to change at a birth weight of approximately 3.4 kg, after which the risk for overweight increased more rapidly with birth weight and decreased more slowly for underweight.

Further adjustment for other maternal or child-related factors, and additional adjustment for maternal height and weight, moderately changed the estimated risks, but the quadratic trend remained unchanged. As compared with a birth weight of 3.00 to 3.24 kg, a birth weight ≥4.25 kg was associated with a relative risk of overweight of 2.17 (95% CI, 1.83–2.59) and a risk of underweight of 0.50 (95% CI, 0.39–0.64) (Table 3). Further analysis stratified by child age at follow-up did not change the patterns for the associations with overweight and underweight (*P* for quadratic trend <0.001) in any stratum.

**Table 3.** Multivariate-adjusted relative risks (RRs) and 95% confidence intervals of overweight and underweight in Chinese children aged 3–6 years

Birth weight (kg)	Overweight			Underweight		
	
	No. of cases	RR_1_^a^	RR_2_^b^	No. of cases	RR_1_^a^	RR_2_^b^
<2.50	88	0.77 (0.61–0.97)	0.80 (0.64–1.01)	513	2.53 (2.27–2.83)	2.31 (2.07–2.58)
2.50–	237	0.77 (0.67–0.89)	0.80 (0.69–0.92)	1005	1.69 (1.56–1.82)	1.56 (1.45–1.69)
2.75–	448	0.86 (0.78–0.96)	0.88 (0.79–0.98)	1379	1.33 (1.25–1.43)	1.28 (1.20–1.37)
3.00–	1713	1.00 (Ref)	1.00 (Ref)	3030	1.00 (Ref)	1.00 (Ref)
3.25–	1259	1.05 (0.97–1.13)	1.03 (0.96–1.11)	1660	0.74 (0.70–0.79)	0.77 (0.73–0.82)
3.50–	1624	1.21 (1.13–1.30)	1.18 (1.10–1.26)	1563	0.63 (0.59–0.67)	0.68 (0.64–0.73)
3.75–	576	1.39 (1.26–1.53)	1.32 (1.20–1.46)	413	0.45 (0.41–0.50)	0.51 (0.46–0.56)
4.00–	473	1.90 (1.70–2.12)	1.78 (1.60–1.99)	286	0.52 (0.46–0.60)	0.60 (0.53–0.68)
≥4.25	162	2.38 (2.01–2.83)	2.17 (1.83–2.59)	72	0.41 (0.33–0.53)	0.50 (0.39–0.64)

*P* for linear trend		<0.001	<0.001		<0.001	<0.001
*P* for quadratic trend		<0.001	<0.001		<0.001	<0.001

^a^RR_1_: Adjusted for residence area (northern/southern); child’s sex (male/female); child’s age at follow-up (40–41, 42–47, 48–53, 54–59, 60–65, 66–71, 72–77, 78–79 months); maternal age at delivery (<22, 22–24, 25–27, 28–30, 31–34, ≥35 years); occupation (farmer/non-farmer); education (high school or above, middle school, primary school or below); folic acid supplementation before or during pregnancy, primiparous (yes/no); child’s gestational age at birth (<37, 37–39, 40–41, ≥42 weeks); child’s body length at birth (<P_5_ (43.0), P_5_ to <P_50_ (50.0), P_50_ to <P_95_ (52.0), ≥P_95_ cm); pregnancy-induced hypertension (yes/no); and maternal weight gain during pregnancy (<P_25_ (8.0), P_25_ to <P_50_ (11.0), P_50_ to <P_75_ (14.0), ≥P_75_ kg).

^b^RR_2_: Further adjusted for maternal height (<P_5_ (1.51), P_5_ to <P_25_ (1.56), P_25_ to <P_50_ (1.59), P_50_ to <P_75_ (1.61), P_75_ to <P_95_ (1.66), ≥P_95_ cm) and weight at first prenatal visit during pregnancy (<P_5_ (43.0), P_5_ to <P_25_ (48.0), P_25_ to <P_50_ (52.0), P_50_ to <P_75_ (56.0), P_75_ to <P_95_ (63.0), ≥P_95_ kg).

### Effect of maternal BMI on the relation of birth weight to overweight

We grouped all subjects by quartiles of maternal BMI, and applied an interaction analysis using a logistic model. We observed significant interaction between maternal BMI and birth weight in the models for overweight (*P* = 0.016) and underweight (*P* < 0.001). The associations of birth weight with these 2 outcomes were consistent in all 4 strata of maternal BMI, although the positive association with overweight was stronger for the 4th quartile, and the negative association with underweight was stronger for the 1st quartile.

## DISCUSSION

In this large population-based prospective cohort, birth weight was positively associated with the risk of overweight and negatively associated with underweight in Chinese children aged 3 to 6 years. The associations could not be explained by other potential confounding factors, including maternal BMI. We observed that the relation curves for both overweight and underweight appeared to be parabolic—the relation curve for overweight resembled the right half of a flat parabolic curve and that for underweight resembled the left half.

Several studies^4^^,^^8^^,^^10^^,^^20^ reported a positive association between birth weight and childhood overweight. However, these studies were not able to control for some potential confounders, such as maternal BMI. Our study found that birth weight was positively and linearly associated with childhood mean height, weight, and BMI, and that the associations remained after extensive adjustment for potential confounders, including maternal BMI, pregnancy-induced hypertension, and maternal weight gain during pregnancy. Maternal BMI,^21^ to a certain extent, indicates genetic potential. Further adjustment for maternal BMI did not change the pattern of the association in the present study, although it mildly attenuated the strength of the association. Similar results were also obtained when either maternal height or weight at first prenatal visit was adjusted for. These findings indicate that a component exists in the association of birth weight with overweight that cannot be accounted for by genetic potential alone.

Some studies^12^ reported that a positive relation between birth weight and overweight only existed in some strata of maternal BMI. However, we observed a positive association in all quartiles of maternal BMI, with a slightly stronger association in the top quartile, which reinforces the hypothesis that birth weight affects early childhood growth partly independently of genetic potential.

Data on the relation of birth weight to underweight are scarce.^22^^,^^23^ There is no doubt that an investigation of this relation would contribute to our understanding of the effects of birth weight on later body growth. We found consistent inverse relations between birth weight and underweight in a number of analyses. These findings are additional evidence that birth weight affects childhood growth. It is reasonable to hypothesize that later development of underweight or overweight may share, to some extent, a common mechanism programmed or triggered in the uterus, although postnatal factors such as nutrition definitely play a role.

Birth weight is determined by genetic factors, prenatal factors (the environment in utero), and their interactions.^24^ Overweight has been found to be associated with genetic factors,^25^ and birth weight and subsequent overweight might share a common genetic component.^11^^,^^26^ In our study, the close relations we observed between birth weight and various childhood anthropometric measures suggest that fetal growth affects childhood growth, but whether genetic or environmental factors in utero play a dominant role cannot be determined. Common mechanisms affecting both birth weight and subsequent overweight include an inborn error in adipose tissue regulation and inherited abnormalities in mechanisms controlling food intake and systemic energy expenditure. This underlying mechanism may explain why faster fetal growth increases the risk of early childhood overweight, whereas slower fetal growth increases the risk of early childhood underweight. However, the development and structure of adipose tissue in humans are poorly understood and thus require further investigation.

This was a large population-based prospective cohort study. Approximately 92% of live-born children were located and followed up. All relevant information such as birth weight and maternal height and weight were measured or collected prospectively. The large sample size allowed us to define more birth weight groups to examine the pattern of association between birth weight and childhood growth. In addition, we adjusted for several factors, such as maternal BMI, pregnancy-induced hypertension, maternal weight gain during pregnancy, and child gestational age and body length at birth. These factors had been recognized as potential confounders, but were not fully accounted for in previous studies.^13^

One limitation of the present study was that we did not collect data on diet or physical activity. Obtaining affordable food is no longer a problem in China due to rapid economic development. Children’s diets are mainly determined by their mothers’ preferences, as influenced by maternal education and occupation. Physical exercise is mainly associated with adult obesity^19^ and was unlikely to substantially affect the results. Another limitation was that information on maternal smoking and alcohol consumption during pregnancy was not collected. National data from China indicate that the prevalences of smoking and heavy alcohol drinking among Chinese women were 3.1%^27^ and 0.8%,^28^ respectively, and the values are likely to be lower for pregnant Chinese women. The third limitation in the present study was a digit preference in the recording of birth weight, especially for 100-gram intervals, although this is unlikely to change the overall results, as the digit preference is assumed to be nondifferential over the 9 birth weight groups.

In conclusion, our findings suggest that higher birth weight increases the risk of childhood overweight, that lower birth weight increases the risk of childhood underweight, and that these results cannot be explained by maternal BMI. Birth weight is linearly correlated with childhood growth, and the curves of the relations of birth weight to overweight and underweight appear to be quadratic.
